# Characterization of Transcription Termination-Associated RNAs: New Insights into their Biogenesis, Tailing, and Expression in Primary Tumors

**DOI:** 10.1155/2018/1243858

**Published:** 2018-04-26

**Authors:** Ilaria Laudadio, Sara Formichetti, Silvia Gioiosa, Filippos Klironomos, Nikolaus Rajewsky, Giuseppe Macino, Claudia Carissimi, Valerio Fulci

**Affiliations:** ^1^Dipartimento di Biotecnologie Cellulari ed Ematologia, Sez Genetica Molecolare, Sapienza Università di Roma, Rome, Italy; ^2^Istituto di Biomembrane e Bioenergetica (IBBE), CNR, Bari, Italy; ^3^Laboratory for Systems Biology of Gene Regulatory Elements, Berlin Institute for Medical Systems Biology, Max-Delbrück Center for Molecular Medicine, Berlin, Germany

## Abstract

Next-generation sequencing has uncovered novel classes of small RNAs (sRNAs) in eukaryotes, in addition to the well-known miRNAs, siRNAs, and piRNAs. In particular, sRNA species arise from transcription start sites (TSSs) and the transcription termination sites (TTSs) of genes. However, a detailed characterization of these new classes of sRNAs is still lacking. Here, we present a comprehensive study of sRNAs derived from TTSs of expressed genes (TTSa-RNAs) in human cell lines and primary tissues. Taking advantage of sRNA-sequencing, we show that TTSa-RNAs are present in the nuclei of human cells, are loaded onto both AGO1 and AGO2, and their biogenesis does not require DICER and AGO2 endonucleolytic activity. TTSa-RNAs display a strong bias against a G residue in the first position at 5′ end, a known feature of AGO-bound sRNAs, and a peculiar oligoA tail at 3′ end. AGO-bound TTSa-RNAs derive from genes involved in cell cycle progression regulation and DNA integrity checkpoints. Finally, we provide evidence that TTSa-RNAs can be detected by sRNA-Seq in primary human tissue, and their expression increases in tumor samples as compared to nontumor tissues, suggesting that in the future, TTSa-RNAs might be explored as biomarker for diagnosis or prognosis of human malignancies.

## 1. Introduction

In the last ten years, the technologies for RNA profiling strongly improved. These advances disclosed pervasive transcription of more than 70% of the human genome, with protein-coding genes accounting for less than 2% of the total RNA [1]. Thus, a dominant portion of the transcribed regions on the human genome originates from non-protein-coding genes (noncoding RNAs (ncRNAs)). Increasing evidences are being obtained, indicating that noncoding RNAs possess essential regulatory functions and could be one of the major contributors to the complex traits of the organisms [2].

Small RNAs (sRNAs) of~ 20–30 nucleotides (nt) constitute a large family of ncRNAs that regulate gene expression. Through base pairing, sRNAs recognize their complementary RNAs as their targets and mediate posttranscriptional and/or transcriptional silencing [3]. The best characterized sRNA classes are microRNAs (miRNAs), small interfering RNAs (siRNAs), and PIWI-interacting RNAs (piRNAs). All these classes of sRNAs are loaded onto a member of Argonaute family proteins. Argonaute proteins typically have a molecular weight of~ 100 kDa and can be divided into two subfamilies: AGO subfamily (e.g., AGO1, AGO2, AGO3, and AGO4 in mammals) that binds to miRNAs and siRNAs and PIWI subfamily (HIWI/PIWIL1, HILI/PIWIL2, HIWI2/PIWIL4, and HIWI3/PIWIL3) that binds to piRNAs [4].

sRNA profiling by next-generation sequencing (sRNA-Seq) is the method of choice for the identification of lowly abundant sRNA classes, in addition to the miRNA, siRNA, and piRNA families. Indeed, sRNAs with varying lengths of between 18 and 200 nucleotides have been reported to be derived from specific genomic regions in higher eukaryotes. At least three classes of sRNAs derived from regions mapping around the 5′ termini of genes have been described: transcription initiation RNAs (tiRNAs) [5], transcription start site-associated RNAs (TSSa-RNAs) [6–8], and promoter-associated small RNAs (PASRs) [9, 10]. The origin and function of these RNAs are uncertain, but preliminary evidence suggests that they are involved in epigenetic control of gene expression. Recently it has been described a new family of sRNAs, termed DNA damage-response RNAs (DDRNAs) [11] or double-strand break- (DSB-) induced RNAs (diRNAs) [12], that are generated at sites of DNA damage and control the DNA damage response.

Finally, sRNA mapping around 3′ termini of genes of higher eukaryotes have also been described, such as termini-associated sRNAs (TASRs) [10, 13], antisense TASRs (aTASRs) [14], and transcription termination site-associated RNAs (TTSa-RNAs) [15]. These sRNA classes can vary in length from 22 to 200 nt and their biological roles and mechanisms of biogenesis remain to be elucidated.

Over the years, sRNAs have become the focus of biomarker research, an approach that has been favorably used in the prediction and early detection of disease and in the investigation of response to treatment for several medical conditions. miRNAs are the most frequently assessed for their potential role as biomarkers, such as in cancer [16], liver [17], and cardiovascular disease [18] among many others. Moreover, dysregulation of other sRNA species such as piRNAs, small nucleolar RNAs (snoRNAs), and small nuclear RNAs (snRNAs) is being found to have relevance to tumorigenesis, neurological, cardiovascular, developmental, and other diseases [19]. Thus, they might be potentially assessed as biomarkers of disease.

Here, we present a comprehensive characterization of TTSa-RNAs [15] in human cells. We show that TTSa-RNAs are present in both AGO1 and AGO2 complexes immunopurified from human cell nuclei. Moreover, TTSa-RNAs can be detected in both soluble and chromatin-associated nuclear extract. Biogenesis of this class of sRNAs is independent of both DICER and AGO2 endonucleolytic activity. TTSa-RNAs show a very strong bias against a G residue in the first position at 5′ end, which is a specific feature of AGO-bound sRNAs and a peculiar oligoA tail at 3′ end. Finally, AGO-bound TTSa-RNAs derive from genes involved in cell cycle progression regulation and DNA integrity checkpoints. Interestingly, this class of sRNAs can be detected in primary human tissues, and their expression increases in tumor samples as compared to nontumor tissues.

## 2. Materials and Methods

### 2.1. Cell Culture and Transfection

HeLaS3 cells were grown in DMEM medium supplemented with 10% (*v*/*v*) fetal bovine serum, 2 mM L-glutamine and penicillin-streptomycin. HCT 116 WT and DICER^Ex5^ cells [20] were grown in McCoy's 5A medium supplemented with 10% (*v*/*v*) fetal bovine serum, 2 mM L-glutamine and penicillin-streptomycin.

HeLaS3 AGO2KO monoclonal cell line was obtained by using two specific Zinc Finger Nucleases (ZNF1 and ZNF2, CompoZr® Knockout Zinc Finger Nucleases, Sigma). HeLaS3 cells were plated in a 6-well plate and transfected using Lipofectamine® 2000 (Thermo Fisher Scientific) with 1.5 *μ*g of each ZNF expression plasmid. After two sequential transfections with the AGO2-specific ZNF expression plasmids, individual clones were isolated by serial dilution and assayed by Western Blot and RT-qPCR for loss of AGO2 expression.

### 2.2. Nuclear Fractionation

Nuclear fractionation was performed similar to [21]. 12 x 10^6^ adherent HeLaS3 cells were washed in 1xPBS and then recovered by scraping and centrifugation. Cell pellets were resuspended in 200 *μ*l of Buffer A (10 mM HEPES pH 7.5, 10 mM KCl, 10% glycerol, 340 mM sucrose, 4 mM MgCl2, 1 mM DTT,1 x Protease Inhibitor Cocktail, Sigma), and then an equal volume of Buffer A with 0.2% (*v*/*v*) Triton X-100 was added and the mixture was incubated on ice for 12 minutes to lyse cells, followed by centrifugation (1200 ×g, 5 min, 4°C). The crude nuclear pellet was resuspended in 300 *μ*l NRB (20 mM HEPES pH 7.5, 50% glycerol, 75 mM NaCl, 1 mM DTT, 1 x protease inhibitor cocktail), transferred to a microcentrifuge tube, and centrifuged (500 ×g, 5 min, 4°C) to wash. The pellet was resuspended in 100 *μ*l NRB, and then an equal volume of NUN buffer (20 mM HEPES, 300 mM NaCl, 1 M urea, 1% NP-40 Substitute, 10 mM MgCl2, 1 mM DTT, 1 x Protease Inhibitor Cocktail) was added and incubated 5 minutes on ice and then centrifuged (1200 ×g, 5 min, 4°C). The soluble nuclear extract supernatant was transferred to another tube, and the depleted nuclear pellet was resuspended in 1 ml Buffer A to wash, transferred to another microcentrifuge tube, and centrifuged (1200 ×g, 5 min, 4°C). Resulting purified chromatin pellets were resuspended in 200 *μ*l Buffer A. TriFast (0.6 ml) (EuroClone) was added to 140 *μ*l of each fraction. As a spike-in control, 2 fmol of a synthetic nonhuman microRNA (ath-miR-159a) was added to each fraction.

### 2.3. RNA Isolation and RT-qPCR

RNA from HeLa S3, HeLa S3 AGO2KO, HCT 116 WT, and DICER^Ex5^ cells, as well as fractions from nuclear fractionation protocol, was isolated using Direct-zol™ RNA MiniPrep Kit (Zymo Research) according to the manufacturer's protocol.

For RT-qPCR of AGO2, ACTB, and MALAT-1, RNA was retrotranscribed using GoScript™ Reverse Transcriptase and random primers (Promega). Quantitative real-time PCR was performed using GoTaq® qPCR Master Mix (Promega). Primer sequences are as follows:

AGO2F ggaggtctgtaacattgtgg, AGO2R gcaatagctttatttcctgccc; ACTB F cctggcacccagcacaat, ACTB R gggccggactcgtcatact; and MALAT-1 F aaagcaaggtctccccacaag, MALAT-1 R ggtctgtgctagatcaaaaggca.

TTSa-RNA (Table 1) expression was checked by using Custom TaqMan® Small RNA Assay (Thermo Fisher Scientific). RNA was retrotranscribed using SuperScript II Reverse Transcriptase (Thermo Fisher Scientific), and qPCR was performed using iTaq Universal Probes Supermix (Bio-Rad). Quantification was normalized to small nucleolar RNA U44 (for total RNA) or to ath-miR-159a spike-in (for SNE and CPE fractions), amplified by TaqMan Small RNA Assay (Thermo Fisher Scientific).

### 2.4. Western Blot

For Western blot analyses, the following antibodies were used: anti-AGO2 (11A9, Millipore), anti-Histone H3 antibody (FL-136, Santa Cruz), anti-BAF155 (Abcam), antibeta Tubulin (Sigma).

### 2.5. Small RNA Sequencing

sRNA-Seq was performed from total RNA isolated from HeLaS3 and AGO2KO HeLaS3 cells by IGA TECH (Udine, Italy). Library was prepared using TruSeq Small RNA Library Preparation Kit (Illumina). Sequencing was performed on HiSeq 2500 in a 50 bp single-read mode 10 million reads/sample. It is worth pointing out that the protocol we applied to generate sRNA libraries only captures 5′ phosphorylated RNA molecules and does not allow cloning of sRNAs harboring a 5′Cap.

### 2.6. TTSa-RNA Data Analysis from Cell Lines

RNA Immunoprecipitation and next-generation sequencing were performed as previously published [6, 22]. NGS data quality was checked using FastQC. tRNA and rRNA genomic coordinates on hg38 genome were retrieved from UCSC RepeatMasker track [23] using Table Browser tool [24]. By using bedtools [25], tRNA and rRNA sequences in FASTA format were retrieved from hg38 genome sequence and used to build a bowtie1 [26] reference index for tRNA and rRNA. miRNA hairpin FASTA sequence was downloaded from miRbase (v21) [27], and human hairpins were extracted. Using bowtie-build, a bowtie1 index was obtained for miRNA hairpins.

Each FASTQ file was processed as follows:

1. Adapter sequences were removed using cutadapt; only reads longer than 17 nt were retained.
2. Adapter-cleaned reads were aligned to the abovementioned miRNA hairpin bowtie1 index using bowtie1 with -n 2 -l 18 options.
3. Reads without any valid alignment in step 2 were aligned to the abovementioned tRNA and rRNA bowtie1 index with -n 2 -l 18 options.
4. Reads without any valid alignment in step 3 were aligned to hg38 human reference with bowtie1 with -n 0 -l 18 -m 1 options. Only reads with a single valid alignment were investigated further.

TTSa-RNAs were defined as those reads aligned in step 4, with at least 1 nt overlapping a 5 nt-long window centered on genomic coordinates of polyA sites (GENCODE v24, [26]). Only polyA sites with a 1 nt length were taken into account in all the analyses (99.8% of the 48535 polyA sites reported by GENCODE v24 annotation).

Within each sample, RPM was defined as the number of reads divided by the total number of reads that gave at least one valid alignment in either step 2, 3, or 4 of the alignment procedure ^∗^ 10^6^.

3′ tails of TTSa-RNAs and miRNAs were determined using Tailor v1.1 [28].

### 2.7. PAR-CLIP Data Analysis

PAR-CLIP libraries were analyzed using the same pipeline outlined above.

### 2.8. Read Coverage Plots

Cumulative coverage of sRNAs giving rise to a single alignment in step 4 was computed using bedtools in a window of 1000 nt centered on GENCODE v24 polyA sites. Each sRNA contributed to the coverage of the closest polyA site only. Three polyA sites (# 605212, 627827, and 582464) lying within 500 nt of loci giving rise to very large numbers of small RNA molecules (not-overlapping any polyA site) were omitted to obtain a clearer picture.

For PAR-CLIP datasets, only reads harboring a single mismatch to the reference genome were used to compute coverage.

### 2.9. GO Term Analysis

Ensembl GENE IDs were retrieved from GENCODE v24 annotation. Genes giving rise to TTSa-RNAs were defined as any Ensembl GENE overlapping a single TTSa-RNA. GO term analysis was performed on DAVID [29] by submitting the list of Ensembl GENE IDs overlapped by TTSa-RNA in each sample. As background, the entire set of Ensembl GENE IDs was used. The reported GO terms were obtained by selecting GO_term_BP_4 in the DAVID output and downloading the full table.

Significantly enriched GO terms (FDR < 0.01) were sorted for decreasing “Fold Enrichment” and the Fold Enrichment was plotted for the top 10 GO Terms.

### 2.10. Correlation of mRNA Expression with TTSa-RNA Counts

mRNA expression in HeLaS3 was retrieved from ENA: EXR352930, EXR352926. Reads were pseudoaligned to the human transcriptome, and transcripts were quantified in transcript per million (TPM) using Kallisto v 0.43.1 [30]. Ensembl GENE IDs corresponding to the Ensembl TRANSCRIPT IDs were retrieved using BioMart, and the TPM for all transcripts of each gene were summed, giving a summarized TPM value for each gene. The genes with TPM below the first tertile were filtered out.

mRNA expression in 44 adrenocortical carcinoma primary samples was retrieved from GEO repository (GSE49278) and belongs to the same samples whose sRNA-seq were used for TTSa-RNA quantification as described below. The Affymetrix Human Gene 2.0 ST Array was analyzed in R (R package version 1.58.1) using Bioconductor packages *oligo* [31] and *genefilter* (using RMA as a background correction/normalization/summarization method). Genes with RMA expression values below the first tertile in more than 10 samples were filtered out; the PROBEIDs were mapped to Ensembl GENE IDs and the Ensembl GENE IDs mapped more than once (<10%) were excluded.

mRNA expression in 10 kidney tumor samples and 10 matched controls was retrieved from SRA project SRP003901 and belongs to the same samples whose sRNA-seq were used for TTSa-RNAs quantification. The digital gene expression (DGE) sequencing reads were trimmed from Illumina adapters using cutadapt [32], resulting in 17 nt reads, which were mapped using bowtie1 with -p 8 -v 0 -m 2 options, after adding the four nucleotides of NlaIII restriction site to the 5′ of each read. The DGE tags belonging to each transcript were quantified using htseq-count [33] (with intersection-strict option) and the htseq-counts were normalized using *DESeq* package [34]. Genes with no counts in more than the 10% of samples and genes with normalized counts below the first tertile were filtered out.

The correlations were computed using custom R scripts and the *R* ^2^ was computed from a Spearman correlation coefficient. In all cases, the genes with TTSa-RNA RPM in the first three quartiles were filtered out, because this corresponded to less than 4 TTSa-RNA reads per sample.

### 2.11. TTSa-RNAs Data Analysis from Primary Samples

A search in SRA was performed using miRNA and cancer/tumor as keywords. We retrieved 17 datasets containing sRNA-seq data for tumor tissue and matched controls. However, only 5 datasets were suitable for our analysis. In fact, we discarded a dataset that was obtained in colorspace (it could not be analyzed using our computational pipeline), a dataset with a sudden drop in Phred quality (median Phred score = 2 for all positions > 16 in all samples), a dataset which contained only one single healthy tissue control sample, three datasets because all reads were shorter than 18 nt (multiple alignments on the genome did not allow specific identification of TTSa-RNAs), and six datasets in which abundant mRNA fragments with random size distribution were retrieved among sRNA-seq reads, suggesting that mRNA degradation does not allow proper identification of bona fide TTSa-RNAs.

The 5 datasets analyzed correspond to SRA projects: SRP028291, SRP014142, SRP045645, SRP003902, and SRP048750. Analysis of these datasets was performed as outlined above for AGO protein-bound sRNAs, except for the application of an 18 to 26 nt size filter as discussed in the results and discussion section. All *p* values were computed using Wilcoxon sum-rank test (a paired version of the test was used for datasets containing paired normal tissue and tumor tissue from each patient) using R function wilcox.test (https://www.r-project.org/).

### 2.12. Availability of Data and Materials

The following datasets analyzed during the current study are available in the ENA repository:

(1) http://www.ebi.ac.uk/ena/data/view/ERX352930

(2) http://www.ebi.ac.uk/ena/data/view/ERX352926

(3) http://www.ebi.ac.uk/ena/data/view/ERX344794

(4) http://www.ebi.ac.uk/ena/data/view/ERX344797

(5) http://www.ebi.ac.uk/ena/data/view/ERX350060

(6) http://www.ebi.ac.uk/ena/data/view/ERX338767

(7) http://www.ebi.ac.uk/ena/data/view/ERX338764

The following datasets analyzed during the current study are available in the SRA repository;

(1) https://trace.ncbi.nlm.nih.gov/Traces/sra/sra.cgi?study=SRP038925

(2) https://trace.ncbi.nlm.nih.gov/Traces/sra/?study=SRP028291

(3) https://trace.ncbi.nlm.nih.gov/Traces/sra/sra.cgi?study=SRP014142

(4) https://trace.ncbi.nlm.nih.gov/Traces/sra/sra.cgi?study=SRP045645

(5) https://trace.ncbi.nlm.nih.gov/Traces/sra/sra.cgi?study=SRP003902

(6) https://trace.ncbi.nlm.nih.gov/Traces/sra/sra.cgi?study=SRP048750

(7) https://trace.ncbi.nlm.nih.gov/Traces/sra/?run=SRR650321

(8) https://trace.ncbi.nlm.nih.gov/Traces/sra/?run=SRR650318

## 3. Results and Discussion

### 3.1. Nuclear AGO1 and AGO2 Bind a Class of sRNAs Arising from Human Transcription Termination Sites

To obtain an overview of nuclear sRNA classes bound to AGO proteins in human cells, we reanalyzed a set of sRNA-seq libraries generated by immunopurifying AGO1 and AGO2 from nuclear extracts of HeLaS3 cell line [6]. We focused on less characterized classes of AGO bound sRNAs, that is reads not aligning to known human miRNAs (miRbase 21) or human tRNAs and rRNAs. All the reads surviving to this preliminary filtering were aligned to human hg38 reference genome requiring unambiguous mapping to a unique genomic position. Reads with multiple mappings on the hg38 reference genome were not further investigated (see Materials and Methods for details).

**Figure 1:**
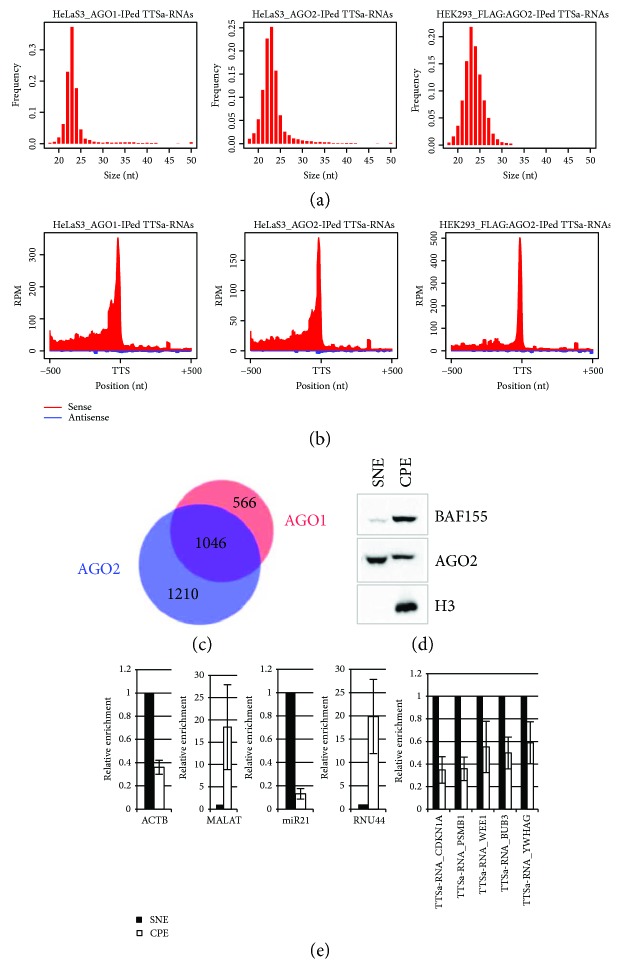
TTSa-RNAs are associated with nuclear AGO1 and AGO2. (b) Coverage of AGO1- and AGO2-immunoprecipitated TTSa-RNAs (HeLa S3) and FLAG:AGO2-immunoprecipitated TTSa-RNAs (HEK293) around GENCODE v25 annotated TTSs. Red and blue represent sRNAs in the sense and antisense orientation with respect to gene transcription, respectively. (c) Overlap of genes giving rise to AGO1- and AGO2-associated TTSa-RNAs. (d) Purified nuclei from HeLaS3 cells were extracted with a forcing urea/detergent buffer to yield a soluble nuclear extract (SNE) and chromatin pellet extract (CPE). SNE and CPE were analyzed by Western blot for the presence of chromatin-associated proteins (BAF-155 and H3) and AGO2. (e) RNA was isolated from HeLaS3 SNE and CPE and analyzed by qRT-PCR for the detection of ACTB mRNA (as a non-chromatin-associated RNA), MALAT-1 (as a chromatin-associated RNA), miR-21, RNU44, and 5 selected TTSa-RNAs (*n* = 3). Synthetic ath-miR159a was added to SNE and CPE fractions before RNA isolation and subsequently used for qRT-PCR normalization.

We found that in nuclei of human cells, both AGO1 and AGO2 bind a class of sRNAs (21–24 nt long, Figure 1(a)) arising from TTSs of human mRNA transcripts and lying on the sense strand of the gene they arise from (Figure 1(b)). Notably, 3′ end of these sRNAs in most cases (60% to 66% depending on the dataset analysed) maps within 2 nt of GENCODE-annotated polyA sites. We assumed that these sRNAs belong to the class of TTSa-RNAs. Indeed, TTSa-RNAs have been described as AGO1/2-associated, miRNA- (22–24 nt) sized sRNAs whose 3′ends aligned with the annotated polyadenylation sites and mapping on the sense strand [15]. Another class of sRNA mapping around 3′ termini of genes has been described in human cells and in plants, namely TASRs [10]. However, human TASRs are more heterogeneous in size as compared to TTSa-RNAs. Moreover, TASRs, whose 3′termini aligned with annotated polyadenylation sites, map antisense to mRNA transcription. In plants, TASRs are 23-24 nt in length and are bound by an AGO protein [13], similar to TTSa-RNAs. However, like human TASRs, plant TASRs seem to be a heterogeneous class mapping on the sense, on the antisense strand or on both strands of protein coding genes. Although we cannot exclude that TTSa-RNAs are a subclass of TASR, because of differences in size, orientation, and mapping with respect to polyadenylation sites, we believe that TTSa-RNAs are a distinct class of sRNAs.

In our experimental design [6], we attained a sequencing depth of 100 million reads per sample, which is substantially higher than the depth employed by Valen and colleagues [15]. This prompted us to investigate peculiar features of this class of sRNAs in order to return a detailed characterization.

The estimated abundance of TTSa-RNAs is about 100 reads per million (RPM) in libraries generated from the nuclear fraction of sRNAs in HeLaS3, reaching about 300 RPM and 150 RPM in AGO1 and AGO2-immunoprecipitated samples, respectively (Figure 1(b)). As shown in Figure 1(c), TTSa-RNAs arise from 2822 genes, and 37% of these genes give rise to TTSa-RNAs that are loaded on both AGO1 and AGO2. More interestingly, GO term analysis suggests that both genes giving rise to AGO1- and AGO2-bound TTSa-RNAs are significantly enriched for genes involved in cell cycle progression regulation and DNA integrity checkpoints (Supplementary Figure 1). A GO term analysis of genes giving rise to IgG-immunoprecipitated sRNAs lying on TTS did not highlight any significant enrichment for the same GO terms (data not shown).

A parallel analysis on a sRNA-seq library obtained by immunopurification of a FLAG-tagged AGO2 complexes from a HEK293 cell line expressing a FLAG-tagged AGO2 [22] yielded similar results, highlighting that TTSa-RNAs are found in different human cell lines (Figures 1(a) and 1(b)).

Not only TTSa-RNAs show a miRNA-like size that is a specific feature for AGO loading but we also evidenced that AGO2 and AGO1 physically interact with this class of sRNA (Supplementary Figure 1A and B) by taking advantage of PAR-CLIP-Seq datasets [35]. Indeed, PAR-CLIP shows that TTSa-RNAs are associated with AGO2 and AGO1. Interestingly, the abundance of TTSa-RNAs in PAR-CLIP libraries is higher compared to the one observed in noncrosslinked AGO IPs, suggesting that TTSa-RNAs are directly associated with AGO proteins. Notably, only reads containing one single mismatch with reference (mainly T->C transitions, Supplementary Figure 1C and D) were used to generate Supplementary Figure 1A and B, thus ensuring that only *bona fide* AGO crosslinked molecules are contributing to the signal observed.

Furthermore, we asked whether TTSa-RNA expression levels are correlated with the abundance of the corresponding mRNA. We looked at the correlation between gene expression levels (as assessed by RNA-seq) of each gene and the abundance of the TTSa-RNAs arising from TTSs of the same gene in HeLaS3 cells for both AGO1 (*R* ^2^<0.1) and AGO2 (*R* ^2^<0.1, *n* = 2). The *R* ^2^ values observed do not support any correlation between gene expression and TTSa-RNA abundance.

Differently from Valen and colleagues [15], we showed that TTSa-RNAs can be detected in libraries obtained from nuclear extracts of HeLaS3 cells. We also investigated whether TTSa-RNAs are recruited on chromatin. We extracted HeLaS3 nuclei to separate soluble and loosely bound material (soluble nuclear fraction (SNF)) from the chromatin pellet (chromatin pellet extract (CPE)), which retains tightly bound factors [21] (Figures 1(d) and 1(e)). As a control of a chromatin-associated RNAs, we measured MALAT [36]. Moreover, in order to provide evidence that this procedure also preserves chromatin localization of sRNAs, we verified that the small nucleolar RNA RNU44 was enriched in the CPE as compared to SNF. On the other hand, we included in the analysis a soluble nuclear-enriched mRNA, beta-actin (ACTB), and miR-21, which has been previously detected not only in the cytosol but also in the nucleus of human cells [37] (Figure 1(e)). We selected 5 abundant TTSa-RNAs arising from genes belonging to the enriched GO categories (*YWHAG*, *WEE1*, *PSMB1*, *CDKNA1*, and *BUB3*, see Table 1). We measured their abundance in different nuclear fractions by qRT-PCR using specific primers designed to selectively amplify sRNA molecules and not their precursors [38]. TTSa-RNAs display a profile of nucleoplasm/chromatin abundance very similar to the one of ACTB mRNA and miR-21 rather than MALAT and U44 (Figure 1(e)), arguing against their specific recruitment on chromatin.

### 3.2. DICER Is Not Involved in TTSa-RNA Biogenesis

**Figure 2:**
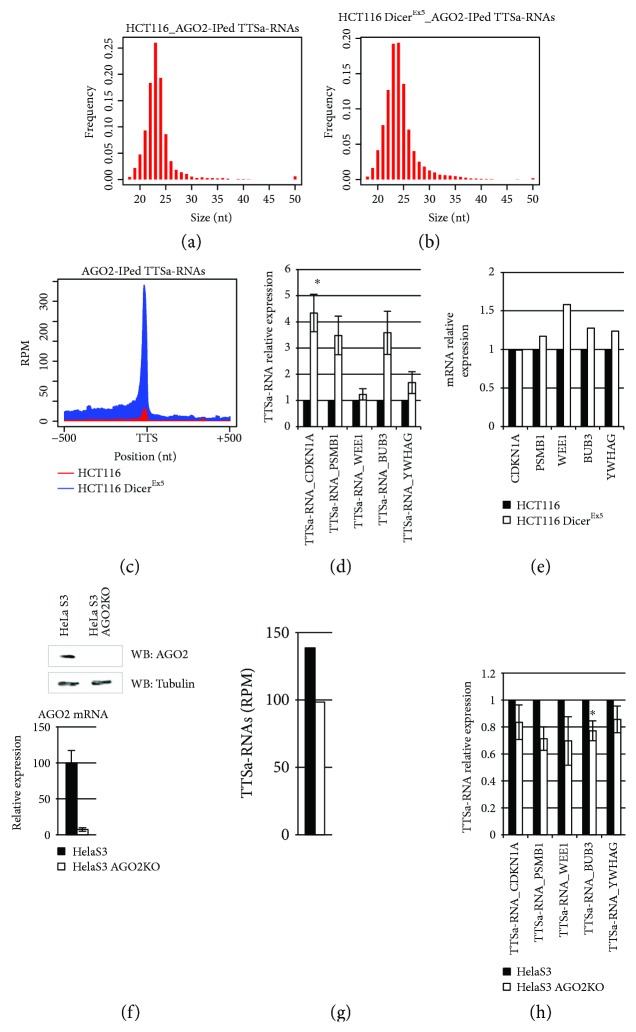
TTSa-RNA biogenesis is independent of both DICER and AGO2. (a) Size distribution of AGO2-immunoprecipitated TTSa-RNAs from HCT116 and HCT116 Dicer^Ex5^ (b) nuclear lysate. (c) Coverage of AGO2-bound TTSa-RNAs around GENCODE v25 annotated human TTSs in parental HCT116 (in red) and HCT116 Dicer^Ex5^ subclone (in blue). (d) qPCR analysis of selected TTSa-RNAs in parental HCT116 and HCT116 Dicer^Ex5^ subclone (*n* = 4). RNU44 was used for normalization. (e) Microarray analysis (GEO dataset GSE6427) of the CDKNA1, PSMB1, WEE1, BUB3, and YWHAG mRNA expression in parental HCT116 and HCT116 Dicer^Ex5^ subclone. (g) Abundance (RPM) of TTSa-RNAs in sRNA libraries generated from whole cell extract of parental HelaS3 and AGO2KO cells. (h) *AGO2* has been genetically knocked out (AGO2KO) in HeLaS3 cells by using specific Zinc Finger Nucleases. In these cells, AGO2 is undetectable at protein level (upper panel) and strongly reduced at mRNA level (lower panel). (f) qPCR analysis of selected TTSa-RNAs in parental HeLaS3 and HeLaS3 AGO2 KO subclone (*n* = 5). RNU44 was used for normalization. qPCR data are expressed as mean ± SEM. (^∗^ *p* value ≤ 0.05; paired *t test*).

We next focused on TTSa-RNAs biogenesis. Previously, it has been speculated that the miRNA biogenesis pathway was not involved in TTSa-RNA processing [15]. We aimed to experimentally verify this hypothesis and we first investigated whether TTSa-RNAs are processed by DICER. To answer this question, we reanalyzed sRNA-seq libraries derived from sRNAs bound to nuclear AGO2 in HCT116 and HCT116 Dicer^EX5^ (a subclone of HCT116 cells in which DICER has been impaired) cell lines [20] (Figures 2(a) and 2(b)) that we previously generated [6]. If DICER endonucleolytic activity is required for TTSa-RNA biogenesis, one would expect a dramatic global reduction of TTSa-RNA abundance as it happens for miRNA [20] and for TSSa-RNAs [6]. On the contrary, we found that AGO2-bound TTSa-RNA abundance is considerably increased in HCT116 Dicer^EX5^ cells compared to parental HCT116 cells (Figure 2(c)). To further validate these findings by qRT-PCR, we analyzed the expression of the same five TTSa-RNAs mentioned above by qRT-PCR in whole cell extracts in HCT116 Dicer^EX5^ cells as compared to parental cells. None of the five TTSa-RNAs tested by qRT-PCR displayed any decrease in HCT116 Dicer^EX5^ cells (Figure 2(d)). In agreement with NGS data, qPCR validation showed that for some of the tested TTSa-RNAs, abundance increases in HCT116 Dicer^EX5^ compared to parental cells. Therefore, in order to exclude that this increase was due to a higher expression of the corresponding gene in HCT116 Dicer^EX5^, we took advantage of the GEO dataset GSE6427 which reports mRNA expression levels in HCT116 and HCT116 Dicer^EX5^ cells. As shown in Figure 2(e), the expression of the YWHAG, WEE1, PSMB1, CDKNA1, and BUB3 mRNAs is not affected by DICER ablation. We hypothesize that higher levels of TTSa-RNAs in DICER hypomorphic cells as compared to control, observed both in NGS data and in qPCR validations, might be explained taking into account that lack of mature miRNA may promote loading on AGO1 and AGO2 of TTSa-RNAs, perhaps increasing their stability. Overall, our data highlights that TTSa-RNA biogenesis is not dependent on DICER.

Since it has been reported that miR-451 is processed in a DICER-independent manner by AGO2 endonucleolytic activity [39], we wondered whether TTSa-RNAs might be processed by AGO2. In case AGO2 was required for TTSa-RNA biogenesis, AGO2 depletion should have resulted in a major decrease of TTSa-RNA abundance in whole cell extracts. We therefore profiled sRNAs in parental and AGO2KO HeLaS3 cells (obtained by genome editing mutation of *AGO2* gene, Figure 2(e)) by sRNA-Seq and we calculated TTSa-RNA abundance (Figure 2(g)). Moreover, we evaluated the abundance of the five selected TTSa-RNAs by qRT-PCR (Figure 2(h)). Both approaches showed a slight decrease in TTSa-RNA abundance when AGO2 is depleted. We concluded that even though AGO2 is not required for TTSa-RNA biogenesis, it might participate to TTSa-RNA stabilization, recapitulating what was previously seen for miRNAs [40].

The fact that both DICER and AGO2 are dispensable for TTSa-RNAs biogenesis is in agreement with our observation that the regions flanking TTSa-RNAs are not biased toward the formation of hairpin structures compared to randomly picked genomic regions (data not shown).

### 3.3. Sequence Characteristics of TTSa-RNAs

**Figure 3:**
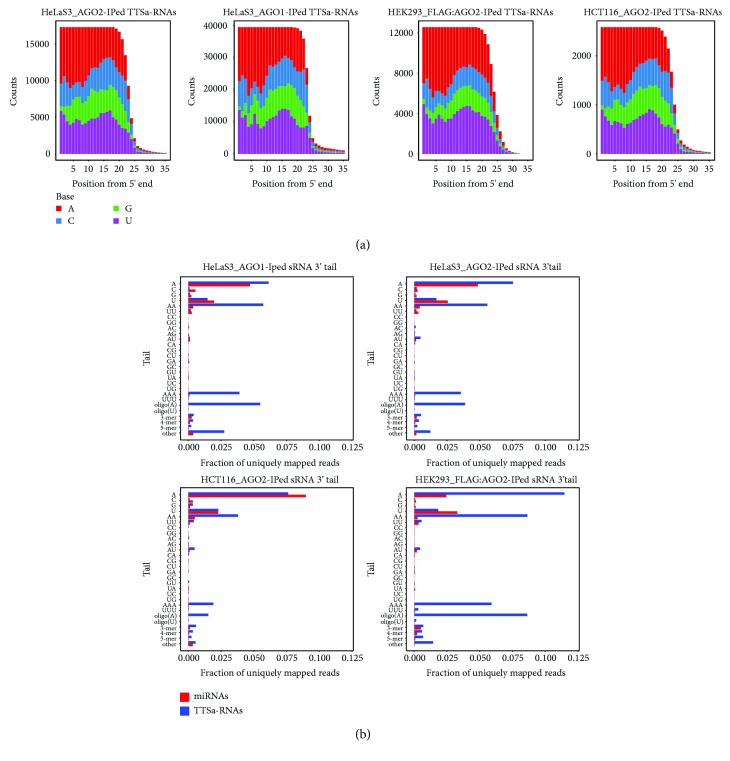
TTSa-RNAs display specific features in 5′ end nucleotide composition and in 3′ end tailing. (a) Nucleotide composition at each position of AGO-bound TTSa-RNAs in different cell lines. (b) 3′ end tail composition and frequency in AGO1- and AGO2-bound TTSa-RNAs (in blue) and miRNAs (in red) from HeLaS3 nuclear extracts, in AGO2-associated TTSa-RNAs and miRNAs in HCT116 nuclear lysate, and in FLAG:AGO2-bound TTSa-RNAs and miRNAs from HEK293T whole cell extracts.

We set out to investigate whether any consensus nucleotide sequence could be found within TTSa-RNAs or in the surrounding genomic sequence. We could not identify a consensus sequence that might provide any hints on TTSa-RNA biogenesis. However, similarly to what was observed for other AGO-bound sRNAs [41, 42], TTSa-RNAs have a bias against a G residue in the first position at 5′ end if compared to other AGO2-bound sRNAs (Fisher Exact test *p* value < 2.2e-16) in all libraries generated from AGO-immunoprecipitated samples (Figure 3(a)). We noted that for all libraries, this bias is stronger in TTSa-RNAs (3.25%) as compared to other AGO-bound sRNAs (8.10%). This can be explained by the low GC content in the regions upstream of human TTSs.

We also checked for any tailing at 3′ end of TTSa-RNAs. We found that about 20% to 27% of TTSa-RNAs bound to endogenous AGO1 and AGO2 displayed a short 3′ tail (1 to 3 nt in most cases). Importantly, 3′ tailing of both AGO1- and AGO2-bound TTSa-RNAs displays a similar pattern. Furthermore, 41% of the TTSa-RNAs bound to FLAG-tagged AGO2 in HEK293T display a 3′ tail (Figure 3(b)). Since it has been reported that miRNAs are 3′ tailed [43, 44], we compared TTSa-RNAs and miRNA 3′ tailing in the same samples. In both sRNA classes, we found abundant monoadenylation and monouridylation. On the other hand, our data highlight a significant (*p* value < 2.2E-16) increase of oligoA tails (4 or more A) in TTSa-RNAs compared to miRNAs. This finding suggests that mature polyadenylated mRNAs might be the precursors of TTSa-RNAs. Although this observation might suggest that TTSa-RNAs are mere degradation by-products of mRNAs, the fact that they arise specifically from the region of mRNAs immediately upstream of polyA sites, the lack of any correlation with corresponding mRNA abundance and the specific loading on AGO proteins argue against this hypothesis. In particular, it is difficult to conceive a general or widespread mRNA degradation pathway leading to the specific accumulation only of degradation fragments flanking polyA sites. Therefore, we hypothesized that these sRNAs are not simply mRNA degradation products but are specific, biologically generated species.

### 3.4. TTSa-RNAs Are Overexpressed in Human Tumors

**Figure 4:**
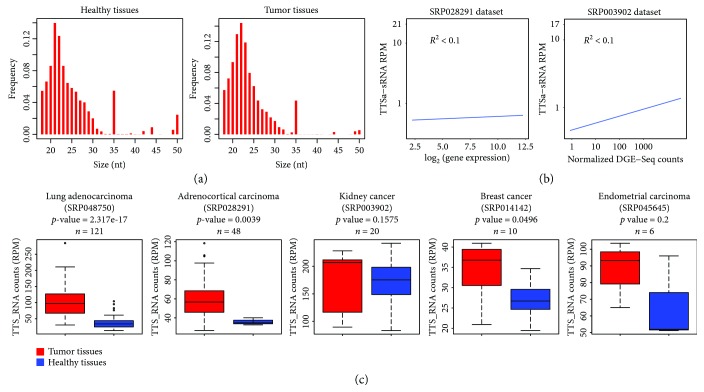
TTSa-RNAs are overexpressed in cancerous tissues compared to matched controls. (a) Size distribution of TTSa-RNAs from primary samples (healthy tissues, left panel; cancerous tissues, right panel). (b) Correlation between mRNA expression level and abundance of TTSa-RNAs in SRP028291 and SRP003902 datasets. Each dot represents a single gene in a single sample. (c) TTSa-RNA expression (RPM) in different tumor types compared to matched control tissues.

Up to now, the existence of sRNAs mapping around 3′ termini of mammalian genes was only reported for cultured cell lines [10, 14, 15]. Therefore, we extended our analysis of TTSa-RNAs to primary samples, and we set out to look for TTSa-RNAs in sRNA NGS libraries obtained from primary human samples. We searched the SRA repository for datasets in which microRNA profile was assessed by NGS. We retrieved five datasets suitable for TTSa-RNA analysis which contained publicly available sRNA expression profiles in tumor samples and matched control tissue. In all the five datasets analyzed, we could detect bona fide TTSa-RNAs displaying the expect size profiles (Figure 4(a)). A small number of reads mapping to TTS with a size of 35 nt were retrieved; therefore, we only counted molecules shorter than 28 nt as bona fide TTSa-sRNAs. Since AGO-bound TTSa-RNAs in cell lines do not correlate with the expression of the corresponding mRNA, we asked whether bona fide TTSa-RNAs that we identified in primary samples display any correlation with mRNA expression. To answer this question, we took advantage of the mRNA expression profiling available for two of the five datasets. As depicted in Figure 4(b), in both cases, we do not observe any significant correlation, highlighting that not only in cultured cell lines but also in human tissues, the amount of TTSa-RNAs is not correlated with the expression of the corresponding gene.

Since deregulation of other sRNA classes has been reported in human tumors [16, 19], we looked at TTSa-RNAs expression in tumor tissues and matched controls. In four out of five datasets analyzed, we found an increase of TTSa-RNAs in tumor samples compared to matched healthy tissues (Figure 4(c)). The lack of statistical significance of the result obtained in endometrial cancer dataset is likely due to the small number of samples in this dataset (*n* = 6). Interestingly, we found that the expression of most TTSa-RNAs is increased in cancer tissues, suggesting a general deregulation of this class of RNAs rather than overexpression of a few TTSa-RNAs (Supplementary Figure 3).

We concluded that TTSa-RNAs are not only detectable in human primary tissues, but their expression also increases in tumors. Accumulation of TTSa-RNAs in tumor samples might reflect the previously reported deregulation of the expression of RNAi components, such as DICER, AGO2, and AGO1 in cancer cells [45]. In particular, downregulation of DICER might impair miRNA processing, thus promoting TTSa-RNA loading on AGO proteins. On the other hand, AGO protein overexpression might stabilize TTSa-RNAs.

## 4. Conclusions

Here, we present a comprehensive study of sRNAs derived from TTS of expressed genes in human cell lines and primary tissues. Taking advantage of a sequencing depth of 100 million reads per sample, this characterization gets insights into previously unknown details of biogenesis, localization, sequence features, and expression of this new and poorly studied class. Our data demonstrate that TTSa-RNAs are a class of DICER-independent and AGO-bound sRNAs, which display an oligoA tailing at 3′ end and which are expressed not only in cultured cell lines but also in human primary tissues. Even though the function of TTSa-RNAs still remains elusive, the fact that genes that give rise to TTSa-RNAs are involved in regulation of proliferation and DNA damage response and that TTSa-RNAs are overexpressed in human tumors suggest that TTSa-RNA expression is linked to tumorigenesis and they might be explored as biomarker for diagnosis or prognosis of human malignancies in the future.

## Figures and Tables

**Table 1:** Sequences of TTSa-RNAs selected for RT-qPCR analysis.

TTSa-RNA	Sequence (5′-> 3′)	Rank of TTSa-RNAs associated to each gene based on their abundance in each library		
		HeLaS3 AGO2-IP	HeLaS3 AGO1-IP	HCT116 AGO2-IP
*BUB3_TTSa-RNA*	CTAATAAACGAGATGCAGAACCCT	4	4	3
*WEE1_TTSa-RNA*	CATATTAAAAGTCACTCTGAGCT	13	13	15
*PSMB1_TTSa-RNA*	TTTATTAAAAGAGAAACCTGAAG	26	39	8
*CDKNA1_TTSa-RNA*	CTCAATAAATGATTCTTAGTGACT	47	32	21
*YWHAG_TTSa-RNA*	CAGTGACGAGGAACTCCCGAGA	86	47	101

## Supplementary Materials

Supplementary Materials Figure 1: AGO proteins are physically associated with TTSa-RNAs. (a) Coverage of AGO2- and FLAG:AGO1-PAR-CLIPPed TTSa-RNAs (HEK293) around GENCODE v25 annotated TTSs. Red and blue represent sRNAs in the sense and antisense orientation with respect to gene transcription, respectively. (b) Mutation frequency of all possible base transitions for AGO2- and FLAG:AGO1-PAR-CLIPPed TTSa-RNAs. Figure 2: top-enriched GO term of genes giving rise to TTSa-RNAs. GO term analysis was performed for genes giving rise to HeLaS3 AGO1- and AGO2-IPed TTSa-RNAs, HCT116 AGO2-IPed TTSa-RNAs, and HEK293 FLAG:AGO2-IPed TTSa-RNAs. Figure 3: most TTSa-RNAs increase their expression in cancer samples. For each TTSa-RNA, the log2 ratio in cancer tissues relative to matched controls was computed. The distribution of the values obtained is depicted for each dataset.
