# Supplementary material for: Characterization of Transcription Termination-Associated RNAs: New Insights into their Biogenesis, Tailing, and Expression in Primary Tumors
Source: Int J Genomics. 2018 Apr 26;2018:1243858. doi: 10.1155/2018/1243858 (PMC5944193; doi:10.1155/2018/1243858)
Supplement: Supplementary Materials — Figure 1: AGO proteins are physically associated with TTSa-RNAs. (a) Coverage of AGO2- and FLAG:AGO1-PAR-CLIPPed TTSa-RNAs (HEK293) around GENCODE v25 annotated TTSs. Red and blue represent sRNAs in the sense and antisense orientation with respect to gene transcription, respectively. (b) Mutation frequency of all possible base transitions for AGO2- and FLAG:AGO1-PAR-CLIPPed TTSa-RNAs. Figure 2: top-enriched GO term of genes giving rise to TTSa-RNAs. GO term analysis was performed for genes giving rise to HeLaS3 AGO1- and AGO2-IPed TTSa-RNAs, HCT116 AGO2-IPed TTSa-RNAs, and HEK293 FLAG:AGO2-IPed TTSa-RNAs. Figure 3: most TTSa-RNAs increase their expression in cancer samples. For each TTSa-RNA, the log2 ratio in cancer tissues relative to matched controls was computed. The distribution of the values obtained is depicted for each dataset. [file 1243858.f1.pptx]

## Slide 1
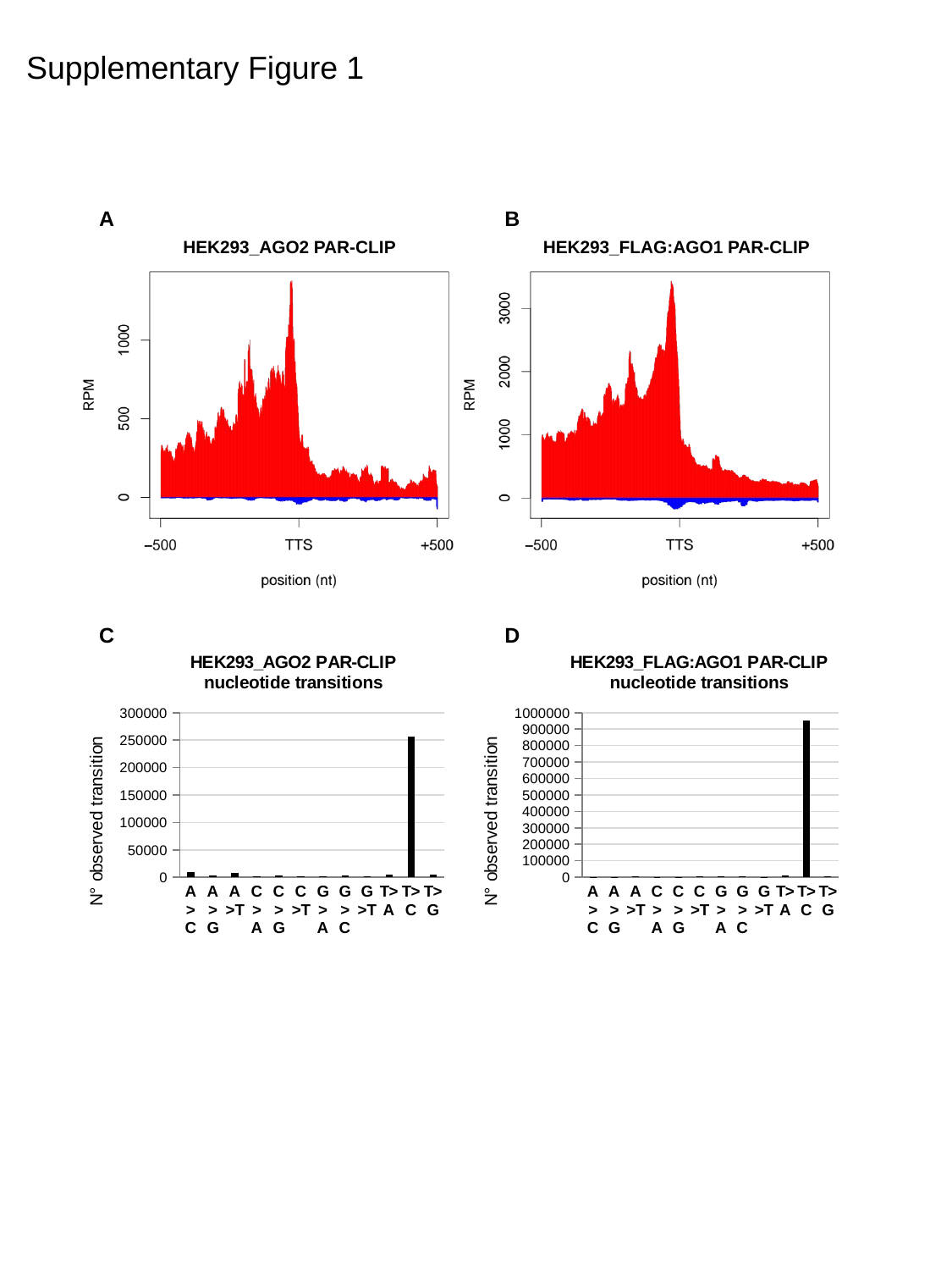

Supplementary Figure 1
A
B
HEK293_FLAG:AGO1 PAR-CLIP
HEK293_AGO2 PAR-CLIP
C
D
### Chart: HEK293_AGO2 PAR-CLIP
nucleotide transitions
| Category | |
|---|---|
| A>C | 9460.0 |
| A>G | 3571.0 |
| A>T | 8628.0 |
| C>A | 1051.0 |
| C>G | 3750.0 |
| C>T | 1949.0 |
| G>A | 2228.0 |
| G>C | 3411.0 |
| G>T | 1510.0 |
| T>A | 4189.0 |
| T>C | 256423.0 |
| T>G | 5454.0 |
### Chart: HEK293_FLAG:AGO1 PAR-CLIP
nucleotide transitions
| Category | |
|---|---|
| A>C | 3008.0 |
| A>G | 3194.0 |
| A>T | 3416.0 |
| C>A | 2119.0 |
| C>G | 1816.0 |
| C>T | 4855.0 |
| G>A | 3611.0 |
| G>C | 3648.0 |
| G>T | 2830.0 |
| T>A | 9356.0 |
| T>C | 952088.0 |
| T>G | 4049.0 |

## Slide 2
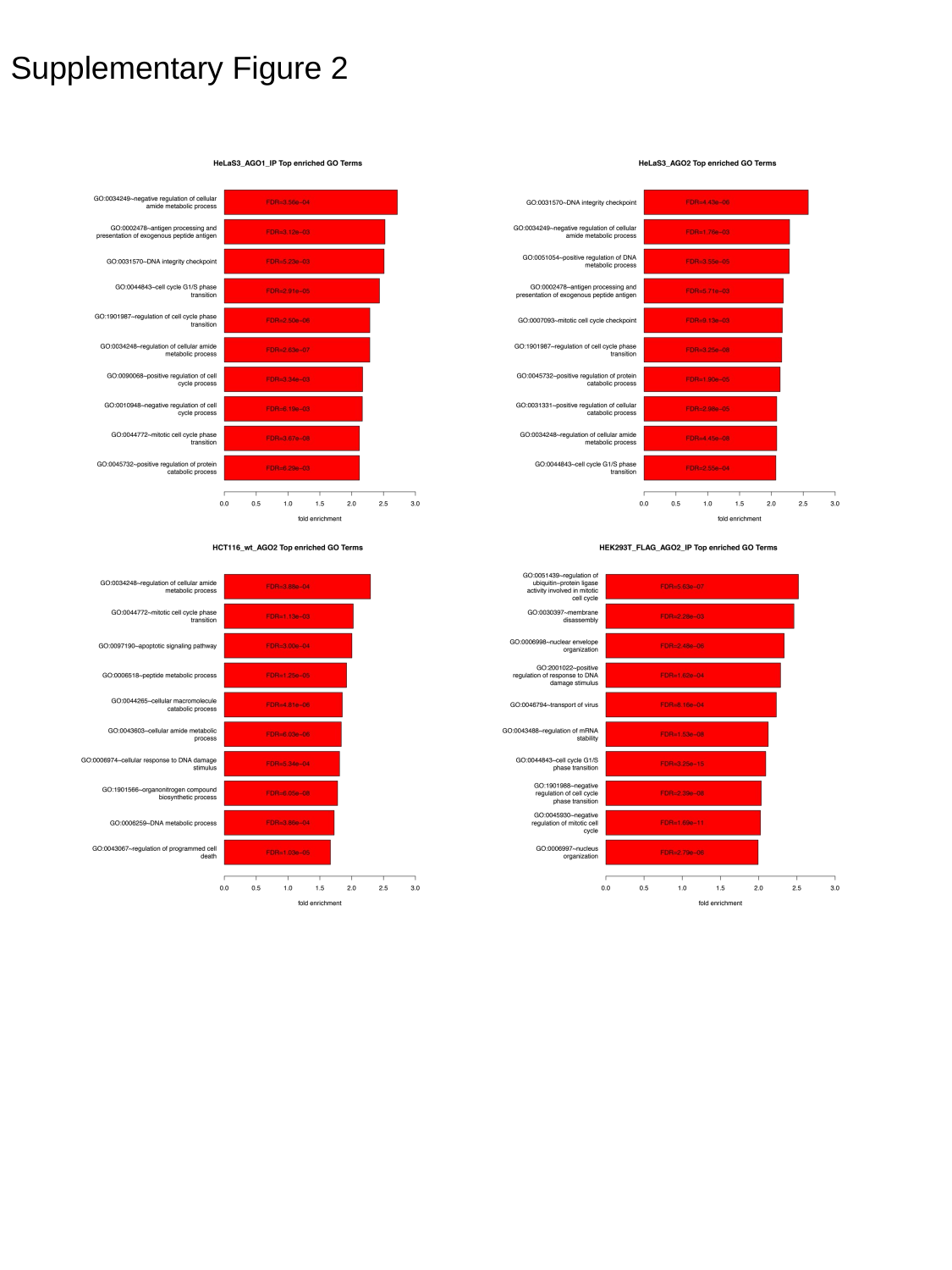

Supplementary Figure 2

## Slide 3
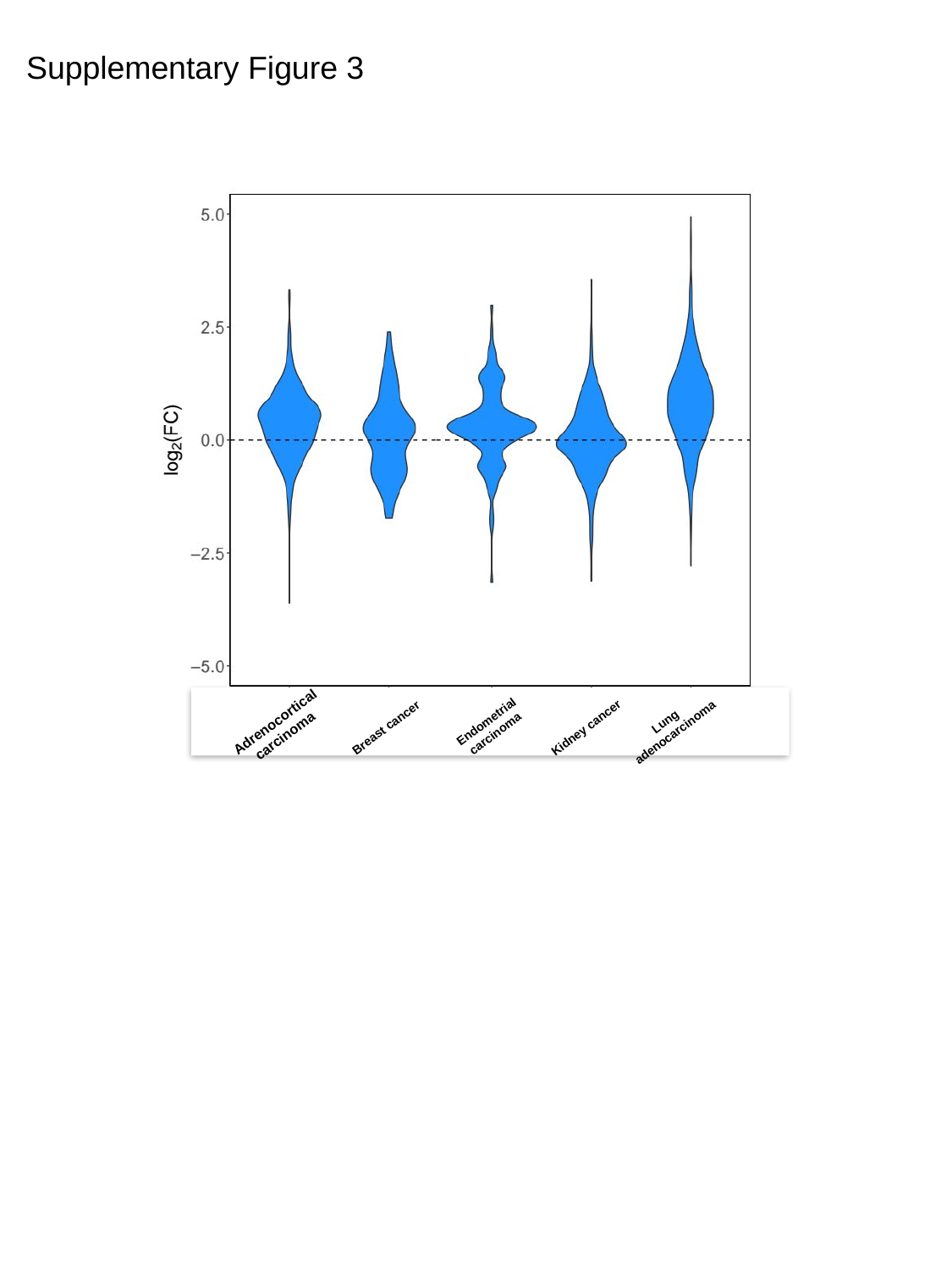

Supplementary Figure 3
Adrenocortical
carcinoma
Endometrial carcinoma
Lung
 adenocarcinoma
Breast cancer
Kidney cancer
